# The need and safety of vitamin supplementation in adults with obesity within 9 months post sleeve gastrectomy (SG): assessment based on intake

**DOI:** 10.1038/s41598-022-18487-z

**Published:** 2022-08-22

**Authors:** Agata Wawrzyniak, Monika Krotki

**Affiliations:** Department of Human Nutrition, Institute of Human Nutrition Sciences, Warsaw University of Life Sciences (WULS-SGGW), Warsaw, Poland

**Keywords:** Nutrition disorders, Obesity

## Abstract

The aim of the study was to assess the need and safety of vitamin supplementation in adults with obesity post bariatric surgery (SG), based on intake assessment. Patients with obesity class III, and with obesity class II with comorbidities were followed up at 3, 6 and 9 months post bariatric surgery. Based on a 4-day food record questionnaire, the intake of vitamins and calories was assessed and an interview regarding the consumption of supplements was conducted. The study showed a deficiency in the dietary intake of vitamin D, folate (B_9_) and vitamin B_1_ (in 93–100% of respondents), vitamins E and C (in 53–67% of respondents), vitamins A, PP and vitamins B_2_ and B_6_ (in 10 to 23% of respondents) and vitamin B_12_ (only 1 woman). The intake of multivitamin supplements was implemented by 72% of respondents, independently, all patients were taking a vitamin D supplement. Vitamin deficiencies were only reported in a small percentage of patients (3–17%), who did not take supplements throughout the observation period. Supplementation with vitamins D, E, C, B_1_ and folic acid (B_9_), used regularly, supplemented the nutritional deficiencies of patients. The intake of vitamin A, B_2_, PP, and B_6_ with supplements did not significantly affect the overall intake. Supplementation with vitamin B_12_ turned out to be unjustified to the nutritional recommendations. The dietary and/or supplemental intake of vitamins did not exceed the tolerable upper intake level (UL). The results of the study confirm the need to implement vitamin supplementation for bariatric patients and its safety.

## Introduction

Obesity is defined as an excessive, pathological accumulation of adipose tissue in the body that leads to the impairment of its functioning^1,2^. According to the World Health Organization, obesity is a chronic disease that poses a threat to the health and life of patients and is treated surgically in the case of obesity class II with concomitant diseases or obesity class III. Currently, the most frequently used type of bariatric surgery is one of the restrictive methods—sleeve gastrectomy (SG)^3^. The anatomical change in the capacity of the stomach accelerates the passage of food, reduces the secretion of the hunger hormone—ghrelin by the stomach, which in turn reduces food intake and aids losing body weight. It may also lead to the disappearance of comorbidities, i.e., cardiovascular diseases, metabolic disorders (including lipid disorders and diabetes), degenerative joint diseases and prolong the life of patients^4,5^. However, due to the restriction of food intake, as well as possible nutritional deficiencies in patients undergoing surgical treatment, dietary counselling plays an important role in the postoperative period, recommending the supplementation of nutrients (including vitamins)^1,2,4,6–10^.

The reason to start the study was a small number of studies analysing the dietary intake of selected vitamins by patients undergoing bariatric surgery^11–13^. The authors of the studies did not analyse the need for supplementation and did not answer the question of whether vitamin supplementation recommended to patients supplemented the deficiencies in the intake of vitamins in the diet. Most of the conducted studies did not assess intake of as many vitamins as in the present study. The aim of this study was, therefore, to assess whether routine vitamin supplementation was necessary and sufficient to prevent deficiencies in vitamin intake in patients undergoing bariatric surgery—sleeve gastrectomy in the late postoperative period, i.e., from 3 to 9 months post procedure, and whether it did not exceed safe levels.

## Methods

The study followed patients of the General, Oncological and Digestive Tract Surgery Department at the Medical Centre of Postgraduate Education at Orlowski Hospital in Warsaw, Poland. The study received the consent of the Bioethical Commission of the Medical Centre of Postgraduate Education (Warsaw, Poland) on 12 April, 2017 (KB-W-382/2017), as well as the individual consent of patients.

### Study participants

The study included patients treated surgically with obesity class III, and with obesity class II with comorbidities, such as heart disease, metabolic disorders, lipid disorders, diabetes, sleep apnea and osteoarthritis. The patients met the following detailed criteria qualifying for the operation: an age range between 18 and 60 years, BMI ≥ 40 kg/m^2^ or BMI 35–39.9 kg/m^2^ in persons additionally experiencing co-morbidities. Exclusion criteria were as follows: inflammatory bowel disease, chronic oesophagitis, gastric and duodenal ulcers constituting a risk of gastrointestinal bleeding, as well as, digestive tract anomalies, severe heart disease and breathing difficulties, alcohol abuse, drug addiction, pregnancy, mental disorders, personality disorders, severe depression, a possible lack of patient engagement in the post-surgical treatment process, an inability to look after oneself and a lack of due medical care from a caregiver post-surgery^3,14,15^. The study included SG patients who participated in the study regularly, i.e. in the 3rd, 6th and 9th month after the procedure (irregular participants, despite being qualified, were not included).

### Procedures

The study followed SG patients at 3-, 6- and 9-months post-surgery. The participants completed a questionnaire on their age, dwelling place, education and job activity. The patients’ dietary intake was assessed with a 4-day food record covering 3 working days and 1 non-working day. Patients reported all consumed products, meals and drinks in grams/millilitres or in household measurements in three study periods. The portion size was defined using photographs of the products and meals^16^. The consumed foods were calculated into energy and nutrient units by Polish developed computer software used to generate national food label composition tables^17^. The vitamin intake (fat-soluble: A, D, E; water-soluble from group B: B_1_, B_2_, PP, B_6_, folate (B_9_), B_12_ and vitamin C) was reported as a mean value of all three stages, for each subject or for a group of women and men.

Throughout the postoperative follow-up period (including visits at 3, 6, 9 months after surgery), all patients received dietary recommendations from a dietitian in the form of oral recommendations, in the form of a brochure or leaflet, and ready-made menus. The usage of dietary supplements was recommended to each patient by the operating surgeon. Patients decided to choose and purchase one of the commercially available supplements. At each control visit, the patients provided the trade name of the dietary supplement they were taking (with a vitamin content close to or slightly higher than the recommended daily intake) and the dose. The researchers, knowing the composition of dietary supplements generally available on the market (from the product label), calculated the amount of vitamins taken by the patient. Patients most commonly took one tablet of the MVMM product daily (Centrum, Vigor, Bodymax) and additionally vitamin D in the amount of 25 µg (1000 IU). The percentage of patients who did not meet the EAR (Estimated Average Requirement) or AI (Adequate Intake) Polish recommendations^18^ was determined during the 3-, 6- and 9-month post-surgery time periods, taking only diet, as well as diet and supplements into account. Taking into account the differences in the bioavailability of food folate and folic acid from dietary supplements, the total amount of these compounds was expressed as the Dietary Folate Equivalent (DFE), where 1 μg DFE = 1 μg diet folate = 0.5 μg folic acid^17^. Moreover, the tolerable upper intake level (UL) was used to verify the safety of the vitamin intake for patients (established UL values: vit. A (RE)—3000 µg/d, vit. D—100 µg/d, vit. E—300 mg/d, vit. C, vit. B_1_ and B_2_—no adequate data to derive a UL, vit. PP (nicotinamide)—700 mg/d, vit. B_6_—25 mg/d, vit. B_9_ (synthetic folic acid)—1000 µg/d, vit. B_12_—no clearly defined adverse effects)^19^. The Tolerable Upper Intake Level (UL) is the maximum level of total chronic intake of a nutrient from all sources judged to be unlikely to pose a risk of adverse health effects in humans.

### Statistical analysis

Statistical analysis was conducted using the IBM SPSS Statistics25 (SPSS Inc, Chicago Ill, USA) software package. Descriptive statistics and distribution normality testing of continuous variables were performed using the Shapiro–Wilk test. The results were presented as mean values, standard deviations and as percentages according to the type of variable. To verify the significance between groups in repeatable measurements, the non-parametric Wilcoxon test (for groups before and after the implementation of supplementation) was used, and in the case of the nominal variable for repetitive measurements—the McNemar test. The need for supplementation was verified using logistic regression analysis. The odds ratio (OR) and 95% confidence intervals (95% CI) were calculated. The reference category was the group without supplementation (OR = 1.00). The value of α = 0.05 was considered as statistically significant.

### Ethical approval and consent to participate

The study was conducted according to the guidelines of the Declaration of Helsinki and approved by the Bioethical Commission of the Medical Centre of Postgraduate Education (Warsaw, Poland) on April 12, 2017 (KB-W-382/2017). Informed consent was obtained from all subjects involved in the study.

## Results

Initially, the study included 43 women and 16 men after SG surgery, but only 30 patients participated systematically in 3 periods, i.e. 24 women (W) aged 44 ± 10 years and 6 men (M) aged 50 ± 7 years (50% of patients). Women and men did not differ significantly regarding age, place of dwelling, education level and job activity. There were no statistically significant differences in energy supply in the 3rd, 6th and 9th month after the procedure between women (average 1084 ± 299 kcal/day) and men (average 1221 ± 279 kcal/day). The intake of dietary supplements remained at a similar level in the period from 3 to 9 months after the surgery, both among women and men (supplements were taken by 70% of women and 78% of men, p > 0.05), which allowed for the assessment of the vitamin intake from the diet, diet and supplements in the aforementioned total period (data published^20^). Independently, all patients took a vitamin D supplement (25 µg/day, i.e., 1000 IU).

**Figure 1:**
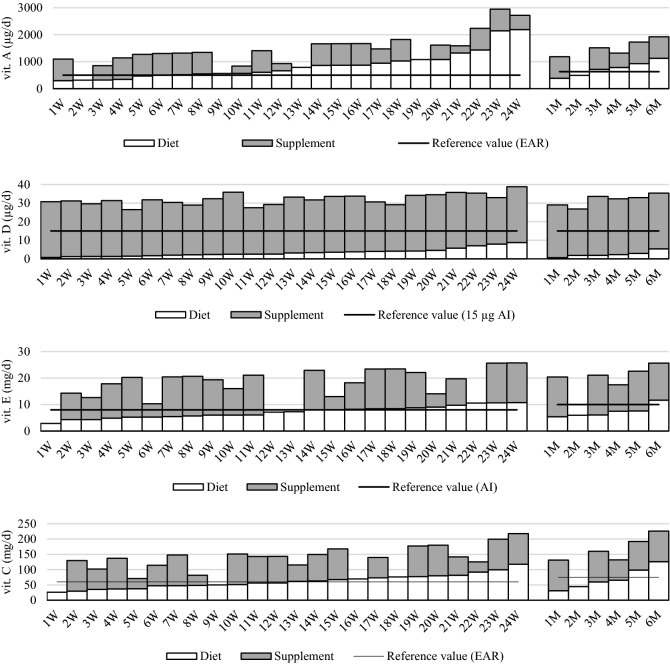
Mean dietary and supplement fat-soluble vitamins (A, D, E) and water-soluble vitamin C intakes by women (**W**) *(n* = *24)* and men (**M**) *(n* = *6)* tested at 3, 6 and 9 months after bariatric surgery (SG) (all data as µg or mg/day).

The dietary intake of fat-soluble vitamins was consistent with the recommendations for vitamin A in 77% of patients and vitamin E in 33% of patients (Fig. 1). Vitamin A supplementation was successfully implemented by 5 patients (supplementing vitamin A deficiencies to the recommended values), and in women the change in the vitamin A intake was statistically significant (p = 0.046) (Table 1). Supplementation with vitamin E turned out to be statistically significant to meet the recommendation for both women (p = 0.001) and men (p = 0.046), and all respondents who implemented supplementation (53% of patients with insufficient dietary intake) achieved an appropriate level of vitamin E intake.

**Table 1:** Mean vitamin dietary intake with or without supplements in patients tested at 3, 6, and 9 months after bariatric surgery (SG).

Vitamin intake	Women *(n* = *24)*		*p* value	Men *(n* = *6)*		*p* value	OR (95% CI)*** p value *(n* = *30)*
	Without suppl	With suppl		Without suppl	With suppl		
Vitamin A*****							
Mean ± sd (µg/day)	847 ± 512	1403 ± 615	< 0.001*	739 ± 273	1361 ± 502	0.034	4.26
Intake from food (%)		27–100			33–100		(0.81–22.53)
Subjects with intake < EAR(%)	20.8	4.2	0.046**	33.3	16.7	0.317	0.088
Vitamin D*****							
Mean ± sd (µg/day)	3.4 ± 2.1	32.0 ± 2.9	< 0.001	2.5 ± 1.6	31.7 ± 3.2	0.026	-
Intake from food (%)		2–24			2–15		
Subjects with intake < AI (%)	100.0	0.0	< 0.001	100.0	0.0	0.014	< 0.001 ****
Vitamin E*****							
Mean ± sd (mg/day)	7.1 ± 2.2	17.0 ± 6.2	< 0.001	7.3 ± 2.3	18.8 ± 6.9	0.039	13.00
Intake from food (%)		26–100			26–100		(3.55–47.60)
Subjects with intake < AI (%)	62.5	12.5	0.001	83.3	16.7	0.046	< 0.001
**Vitamin C**							
Mean ± sd (mg/day)	62 ± 23	127 ± 47	< 0.001	71 ± 35	148 ± 62	0.039	10.29
Intake from food (%)		23–100			24–100		(2.56–41.37)
Subjects with intake < EAR (%)	50.0	8.3	0.002	66.7	16.7	0.083	0.001
**Vitamin B**_**1**_							
Mean ± sd (mg/day)	0.71 ± 0.19	1.67 ± 0.57	< 0.001	0.80 ± 0.18	1.87 ± 0.58	0.039	91.00
Intake from food (%)		27–100			29–100		(15.36–539.26)
Subjects with intake < EAR (%)	91.7	12.5	< 0.001	100.0	16.7	0.025	< 0.001
**Vitamin B**_**2**_							
Mean ± sd (mg/day)	1.22 ± 0.32	2.41 ± 0.77	< 0.001	1.39 ± 0.25	2.73 ± 0.81	0.039	3.22
Intake from food (%)		35–100			40–100		(0.32–32.89)
Subjects with intake < EAR (%)	12.5	4.2	0.157	0.0	0.0	1.00	0.324
Vitamin PP*****							
Mean ± sd (mg/day)	13.7 ± 3.1	27.3 ± 8.3	< 0.001	13.7 ± 3.3	29.0 ± 9.8	0.039	4.26
Intake from food (%)		31–100			34–100		(0.81–22.53)
Subjects with intake < EAR (%)	16.7	4.2	0.083	50.0	16.7	0.157	0.088
**Witamina B**_6_*****							
Mean ± sd (mg/day)	1.46 ± 0.33	2.83 ± 0.86	< 0.001	1.70 ± 0.37	3.22 ± 1.04	0.039	3.50
Intake from food (%)		32–100			41–100		(0.65–18.98)
Subjects with intake < EAR (%)	20.8	4.2	0.046	16.7	16.7	1.00	0.146
**Folate (B**_**9**_)*****							
Mean ± sd (µg/day)	159 ± 43	395 ± 142	< 0.001	171 ± 38	436 ± 156	0.034	95.29
Intake from food (%)		22–100			29–100		(10.93–830.86)
Subjects with intake < EAR (%)	100.0	25.0	< 0.001	100.0	16.7	0.025	< 0.001
**Vitamin B**_**12**_							
Mean ± sd (µg/day)	3.91 ± 1.79	5.52 ± 2.03	< 0.001	3.30 ± 0.96	5.25 ± 1.78	0.034	1.00
Intake from food (%)		45–100			53–100		(0.06–16.76)
Subjects with intake < EAR (%)	4.2	4.2	1.00	0.0	0.0	1.00	1.00

*Wilcoxon test; **McNemar test; ***odds ratio (95% confidence interval) for subject intake > EAR/AI with vs. without supplementation.

p ≤ 0.05 statistically significant difference; EAR/AI ref. value, ****—a perfect fit was obtained in the model, *****—the dietary and/or supplemental intake of vitamins did not exceed the tolerable upper intake level (UL).

In the case of vitamin D, only supplementation allowed all patients to intake this nutrient as recommended. The diet did not provide vitamin D in the amounts recommended for any of the patients.

**Figure 2:**
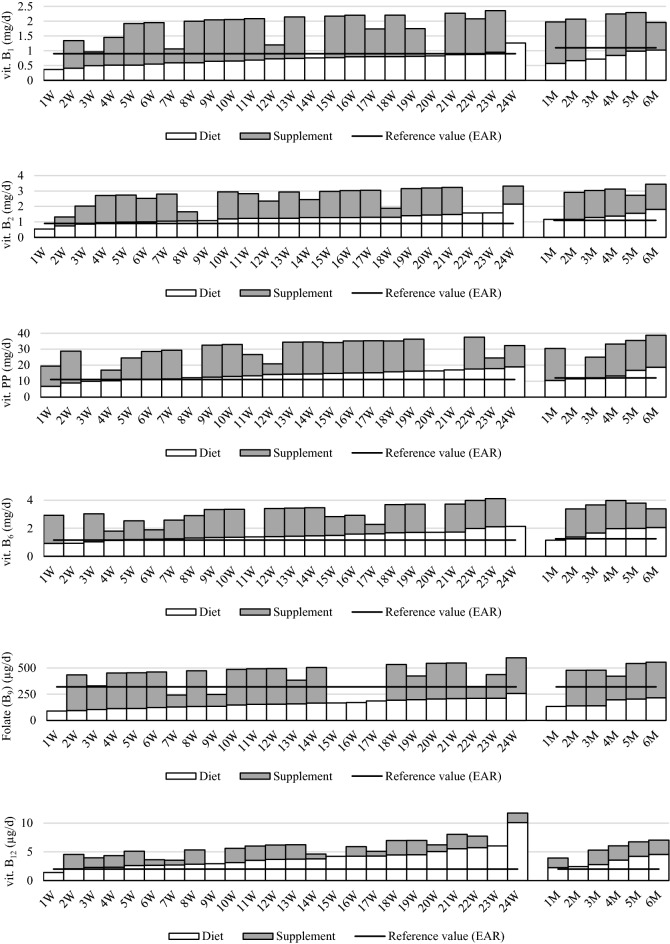
Mean dietary and supplement water-soluble vitamins B intakes by women (**W**) *(n* = *24)* and men (**M**) *(n* = *6)* tested at 3, 6 and 9 months after bariatric surgery (SG) (all data as µg or mg/day).

In the case of water-soluble vitamins, folates (in 100%), vitamin B_1_ (in 93% of patients), vitamin C (in 53% of patients) were rated as the most deficient in the diet, and supplementation turned out to be necessary and statistically significant for the intake of these vitamins, although not all patients implemented it (Fig. 2). Supplementation with folic acid improved compliance with the recommendation in 23 patients (77% of the respondents), taking vitamin B_1_ with the supplement improved the intake in 24 patients (80% of the respondents), and vitamin C in 13 patients (43%). A smaller percentage of patients required supplementation with vitamin PP (23% of respondents), vitamin B_6_ (20% of patients), vitamin B_2_ (13% of women), and taking vitamin supplements did not improve the percentage of patients who followed the dietary recommendations in the study group (OR at p > 0.05). Supplementation with vitamin B_12_ was only required by 1 woman who did not take the vitamin preparation, and supplementation with this vitamin in 97% of patients turned out to be unjustified in relation to the nutritional recommendations. All patients taking the supplements consumed vitamins in amounts exceeding nutritional recommendations, except for 2 women for whom folic acid supplementation was too low due to an irregular intake. Vitamin deficiencies were found in only 3 to 17% of patients who did not implement supplementation.

The intake of vitamin D from supplements (in 100% of patients), vitamins E, B_1_, PP and folates (in 70–73% of patients), vitamins B_2_ and B_6_ (in 57–60% of subjects), vitamin A (in 33% of subjects), was higher than the intake of these nutrients from the diet, however, the intake of any of the vitamins did not exceed the safe UL value. Vitamin B_12_ in an amount greater than from the diet was only taken by 1 person.

## Discussion

The main observations of the study revealed that: (1) the limited energy intake after sleeve gastrectomy is associated with irregularities of vitamin intake and patients require supplementation, as indicated by the Food Pyramid developed by Moizé et al.^21^; (2) the applied supplementation was adjusted to the required intake of vitamins in patients; none of the patients exceeded the UL intake of vitamins with diet and supplements.

Only a few studies document dietary vitamin intake in patients after bariatric SG surgery. An insufficient dietary intake of vitamin A/retinol by patients with obesity was documented in both women and men after SG surgery^11–13^. Vitamin A deficiencies were revealed in the studies of Hosseini-Esfahani et al.^12^ one year after SG surgery (in 96% of patients) and Jastrzębska-Mierzyńska et al.^13^ within 3 and 6 months after SG surgery in selected patients. In a study by Chou et al.^11^, patients did not intake the recommended amount of retinol 5 years after SG surgery. Insufficient dietary fat intake may be considered as the cause of vitamin A deficiency^13^. Although the clinical consequences of dietary vitamin A deficiency following bariatric surgery are rare, they should primarily be suspected in patients who develop ophthalmic complications, such as unexplained visual disturbances or night blindness. In this study, it was observed that patients who implemented supplementation at a level of 800 µg/day supplemented intake deficiencies.

The main source of vitamin D in the human body is skin synthesis under the influence of solar (ultraviolet) radiation. Dermal synthesis contributes to about 90%, and food provides 10% of the daily dose of vitamin D. Vitamin D absorption takes place in the jejunum and ileum^7,22^, therefore, patients after restrictive bariatric surgery (SG) are less exposed to deficiencies of this vitamin. According to scientific societies involved in establishing treatment guidelines after bariatric surgery (American Association of Clinical Endocrinologists (ASCE) / The Obesity Society (TOS)/American Society for Metabolic & Bariatric Surgery (ASMBS), the recommended dose of vitamin D_3_ orally is 75 µg (3000 IU) daily or 1–3 times a week 1250 µg (50,000 IU) of vitamin D_2_^23–25^. The size of the dose should be modified depending on the level of 25(OH)D in the blood serum (with the optimal level of 25(OH)D—30–50 ng/ml)^25–27^. Interestingly, several studies did not recommend vitamin D supplementation, and the body's supply of this vitamin was adequate with weight loss^27,28^, which should be interpreted as the release of vitamin D from resources in adipose tissue. In the conducted studies^12,13^, researchers presented a deficient dietary intake of vitamin D in all subjects, similar to our studies. Supplementation in the amount of at least 25 µg/day used in our study in all the studied patients supplemented the diet intake to an adequate amount.

Vitamin E is classified as an antioxidant, hence the clinical significance of its deficiency. There are no studies describing the clinical symptoms of vitamin E deficiency in patients after SG. Few studies assessing dietary vitamin E intake in patients undergoing SG bariatric surgery^11–13^ have shown a deficiency of vitamin E intake in all subjects within 3 months after surgery and in all men within 6 months after surgery^13^, as well as a deficit in dietary intake in 80% of respondents within one year after SG surgery^12^, or in selected patients 5 years after surgery^11^. A dose of 10–15 mg of vitamin E taken daily with supplements in our studies significantly improved vitamin E intake in patients who consumed too little of this vitamin in their diets, supplementing the deficiencies in all of them.

Another vitamin with antioxidant properties is vitamin C. The main sources of vitamin C are vegetables and fruits, which are restricted in patient consumption due to increased fiber content. In the conducted studies^13^, all patients 3 months after SG surgery showed a deficiency in vitamin C intake in the diet, as well as all men in the 6th month post-surgery. Also, in a study conducted one year after surgery, 83% of respondents were deficient in vitamin C intake^12^, similar to the studies by Chou et al.^11^. In our study, all patients with a deficient dietary intake of vitamin C who implemented supplements (with the content of vitamin C in supplements being equal to 60–100 mg) consumed vitamin C in the above recommended amounts, and supplementation significantly increased this intake.

The most deficient vitamins from group B in the studied patients were vitamin B_1_ (in 93% of respondents) and folates (in 100%), and the implemented supplementation significantly improved their intake in the diet, which was not observed in the case of other vitamins, where deficiencies in dietary intake were significantly smaller.

Vitamin B_1_ resources in the human body are sufficient for about 3–6 weeks^29,30^. Vitamin B1 deficiency after bariatric surgery may be caused by rapid weight loss, reduced food intake, decreased gastric acid secretion, persistent vomiting and malabsorption syndrome^25^. Thiamine deficiency was most commonly found in patients after mixed surgery, but it also happens after strictly restrictive surgery. In most cases, supplementation in the form of a multivitamin supplement was sufficient, and patients with vomiting, rapid weight loss and alcoholics required an additional supply of vitamin B_1_^25,31^. In the previously published studies, when assessing the regularity of dietary vitamin B_1_ intake in patients undergoing bariatric SG surgery^11–13^, a deficiency of vitamin B1 intake was found in all subjects within 3 months post-surgery and in all men within 6 months post-surgery^13^, a nutritional deficit in 84.4% of subjects within one year after SG surgery^12^, as well as in subjects 5 years after surgery^11^. In our study, supplementation with vitamin B_1_ in the amount of 1.4 mg a day was sufficient and significantly supplemented the deficiencies in all patients, even if it was taken irregularly.

Folate deficiency is most often a result of limited food intake, mainly leafy green vegetables, wholemeal products, legumes and fruit. The resources of folic acid in the human body are insignificant and are depleted after 2–3 weeks, hence the need for the constant intake of folate in the diet^29,31^. In the studies of other authors^11–13^, similarly to the authors' study, a deficiency of folate intake in the diet was noted in 90–100% of respondents. In our study, the dose of folic acid 200 µg/day in supplements turned out to be sufficient to meet nutritional recommendations.

All researchers agree that supplementation after SG is necessary because the diet did not meet the nutritional recommendations for vitamins, most often vitamin D and folates^22,25,28,32,33^. Controversies concern the duration of supplementation use. Ruiz-Tovar et al.^27^ believe that supplementation after SG is necessary for a period of 3 months and deficiencies are most often related to vitamin D. Most researchers suggest a systematic supplementation according to ASMBS guidelines up to 1 year after surgery^10^.

The strength of this study is the analysis of dietary vitamin intake by patients after bariatric SG surgery and the assessment of the need for supplementation. The studies conducted so far have not assessed whether the recommended vitamin supplementation covers the intake deficiencies up to the recommended values. None of the studies covered such a broad range of vitamins and long time period (3–9 months) post-surgery. The conducted studies did not assess whether the scope of the administered supplementation did not exceed safe UL levels. Nevertheless, our study is subject to several limitations. The study was conducted in a small group of men compared to women, which reflects the fact that women constitute the majority of patients undergoing obesity surgery^12,13,34,35^. The second important limitation is that the authors did not determine the level of vitamins in the blood of the patients. Another limitation was finishing the study 9 months after surgery, since fewer women and no men had a nutrition follow up after 9 months. However, the results of this study confirm that supplementation with selected vitamins, especially vitamin D, E, C, B_1_ and folate, after SG surgery is necessary and safe, and patients require the care of a dietitian to prevent nutritional deficiencies.

## Conclusions

The limited caloric content of the diet after sleeve gastrectomy is associated with the occurrence of nutritional deficiencies of vitamins in patients requiring supplementation, after an individual dietary assessment. Supplementation with vitamins D, E, C, B_1_ and folates, used regularly, was at an appropriate level and significantly supplemented the nutritional deficiencies of the patients. The intake of vitamins A, B_2_, PP, B_6_ with supplements did not significantly affect the overall intake. Supplementation with vitamin B_12_ turned out to be unjustified to the nutritional recommendations. The intake of vitamins in a diet and supplements in none of the patients exceeded the safe UL value.

## Data Availability

The datasets are available on reasonable request from the corresponding author.
